# Clean air for a sustainable world

**DOI:** 10.1038/s41467-021-25885-w

**Published:** 2021-10-04

## Abstract

Air pollution is a cause of disease for millions around the world and now more than ever urgent action is required to tackle the burden of its impacts. Doing so will not only improve both life expectancy and quality of life, but will also lead to a more just and sustainable world.

Recently, we announced that we will publish a new series of collections focused on issues related to the Sustainable Development Goals (SDGs). We start this series with a multidisciplinary collection on air pollution. As tackling air pollution is not one of the core SDGs, this may seem like an unusual choice. It is, however, a pressing environmental hazard affecting an ever increasing part of the world’s population. Currently, 91% of the world’s population live in locations where pollution levels exceed WHO guidelines, and in a recent announcement the WHO have further cut the recommended limits. Air pollution kills around 6.7 million people per year mainly through respiratory and cardiovascular diseases^1^, and has significant impacts on mental health. The main pollutants are sourced from fossil fuel combustion for transport, industry, agriculture and cooking stoves and, therefore, air pollution is linked directly with fulfilling many of our basic needs. As the SDGs aim to tackle the issue of how humanity can live sustainably, it is thus no surprise that addressing air pollution is related to the SDGs in many different ways. Promoting specific SDGs will lead to improved air quality as a side-effect, while reducing emissions will also progress a number of SDGs directly.

The high air pollution levels that we live with today is another demonstration of how our unsustainable lifestyles are one of the key challenges that needs to be overcome to create a more just and liveable world, which is the ultimate goal of the SDGs.

**Figure:**
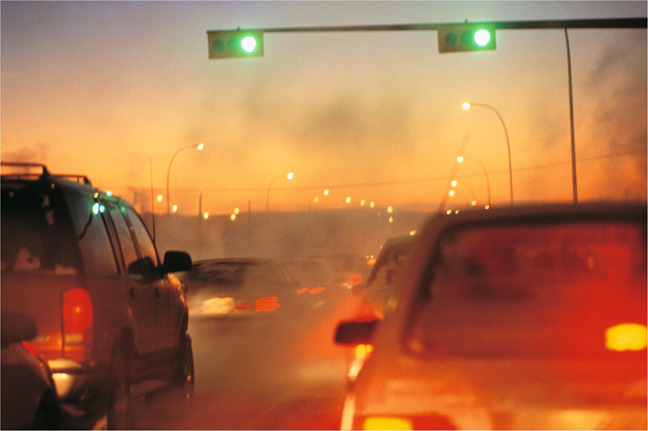
DIGITAL VISION

Although air pollution is a global issue, exposure is often not distributed equally. Industrial processes related to the production, trade and consumption of goods is a key source of air pollution. Much of this pollution is released in low- and middle-income countries while they manufacture goods that are traded abroad, allowing rich countries to outsource the air pollution and health effects of their consumption. Hence, global implementation of responsible consumption and sustainable production practices—the focus of SDG9 (“Industry, Innovation and Infrastructure”) and SDG12 (“Responsible Consumption and Production”)—will be key to reduce this unequal responsibility and exposure to dangerous environmental conditions.

Inequality in exposure does not only occur at an international level, but also within countries. Systematic and historical forms of discrimination often translate into higher exposure levels and, hence, enhanced health burdens to marginalized groups around the world. This is probably best studied in the US, where people of colour are shown to live under poorer air quality, independent of other factors like income^2^. In a commentary for our collection Viniece Jennings highlights that whilst green infrastructure has the potential to reduce air pollution, unequal access can limit improvements for marginalised communities^3^. While we often think of air pollution as an outdoor issue, much of the exposure to harmful particles actually happens inside houses. Household air pollution is mainly related to cooking, heating or lighting, often through the combustion of solid fuels. This exposure affects women and children disproportionately, especially in the developing world^4^. Consequently, targeting SDG10 (“Reduce inequality within and among countries”) and SDG 7 (“Ensure access to affordable, reliable, sustainable and modern energy for all.”) will be of vital importance to tackle embedded inequalities within and among countries to reduce air pollution exposure.

Air pollution and climate change are closely intertwined as they share the same root cause of human emissions. Even though ambitious climate mitigation policies do not come for free, they will in many cases also lead to improved air quality and lower health costs. The societal costs of air pollution avoided through reduced exposure levels as a result of climate mitigation measures alone are thought to outweigh the initial costs of these policies^5^. Air pollution also physically interacts with the climate system; particles in the atmosphere affect surface temperatures as well as clouds and precipitation. Climate change thus has the potential to “worsen air pollution, even in areas where it has been improving”, as pointed out by Denise Mauzarell in a Q&A for our Clean Air collection^6^. An example of this are the dangerous pollutants released by wildfires that are expected to become ever more frequent and intense in many parts of the world.

Similarly, to climate mitigation, improving air quality depends on strict and ambitious regulatory policies and controls, which must be implemented equitably. In this regard, there are reasons to be optimistic, as strict air quality policies like the Clean Air Act in the US and similar policies in Europe have resulted in reductions in pollution since the 1970s even though levels are still too high and continued efforts are crucial. These efforts show that ambitious policy supported by technological advances like improved filtering and modernization can be successful. These efforts should not only be done at national levels, but also need international collaboration, technology and knowledge transfer in order to acknowledge the shared responsibilities of air pollution. As part of the Clean Air collection we highlight papers Nature Communications has published that look at how policy and technology can be part of the solution to air pollution.

The high air pollution levels that we live with today is another demonstration of how our unsustainable lifestyles are one of the key challenges that needs to be overcome to create a more just and liveable world, which is the ultimate goal of the SDGs. Of course, reducing air pollution on its own will not meet the aims of all the other SDGs. Still, it is an illustrative example of how an interdisciplinary focus on a measurable and technologically approachable issue can help to also achieve other goals. It is in this spirit that our collection brings together research from different disciplines, such as applied scientists, economists, political scientists, health scientists and climate scientists as it is this interdisciplinary collaboration that *Nature Communications* wants to support will be vital in informing policy and decision makers. We envision that our collection on Clean Air will continue to grow and we welcome submissions across disciplines in this area.
